# Validation of the Excited Component of the Positive and Negative Syndrome Scale (PANSS-EC) in a naturalistic sample of 278 patients with acute psychosis and agitation in a psychiatric emergency room

**DOI:** 10.1186/1477-7525-9-18

**Published:** 2011-03-29

**Authors:** Alonso Montoya, Amparo Valladares, Luis Lizán, Luis San, Rodrigo Escobar, Silvia Paz

**Affiliations:** 1 Lilly Research Laboratories, Avenida de la Industria 30, 28108 Alcobendas, Madrid, Spain; 2 Outcomes'10, Ronda Mijares, 71 Castellón, Spain; 3 Psychiatry Service, San Igualada Hospital, Passeig Vall d'Hebron 107, 08035 Barcelona, Spain; 4 EU Medical, Lilly Research Laboratories, Avenida de la Industria 30, 28108 Alcobendas, Madrid, Spain

## Abstract

**Background:**

Despite the wide use of the Excited Component of the Positive and Negative Syndrome Scale (PANSS-EC) in a clinical setting to assess agitated patients, a validation study to evaluate its psychometric properties was missing.

**Methods:**

Data from the observational NATURA study were used. This research describes trends in the use of treatments in patients with acute psychotic episodes and agitation seen in emergency departments. Exploratory principal component factor analysis was performed. Spearman's correlation and regression analyses (linear regression model) as well as equipercentile linking of Clinical Global Impression of Severity (CGI-S), Agitation and Calmness Evaluation Scale (ACES) and PANSS-EC items were conducted to examine the scale's diagnostic validity. Furthermore, reliability (Cronbach's alpha) and responsiveness were evaluated.

**Results:**

Factor analysis resulted in one factor being retained according to eigenvalue ≥1. At admission, the PANSS-EC and CGI-S were found to be linearly related, with an average increase of 3.4 points (p < 0.001) on the PANSS-EC for each additional CGI-S point. The PANSS-EC and ACES were found to be linearly and inversely related, with an average decrease of 5.5 points (p < 0.001) on the PANSS-EC for each additional point. The equipercentile method shows the poor sensitivity of the ACES scale. Cronbach's alpha was 0.86 and effect size was 1.44.

**Conclusions:**

The factorial analyses confirm the unifactorial structure of the PANSS-EC subscale. The PANSS-EC showed a strong linear correlation with rating scales such as CGI-S and ACES. PANSS-EC has also shown an excellent capacity to detect real changes in agitated patients.

## Background

Agitation and aggressive behaviour due to primary psychiatric disturbances are particularly prevalent in emergency psychiatric services and specialist psychiatric units for acute psychoses [1]. During these emergency situations, some injuries to both patients and staff may occur, and rapid and effective action is required to minimize the risks [2]. A series of instruments are used in clinical and research settings, allowing the rapid assessment of the levels of aggression and anxiety in patients. The preferred measure in modern trials is a subset of items derived from the Positive and Negative Syndrome Scale (PANSS) [3]. PANSS specifically assesses both positive and negative symptoms of schizophrenia as well as general psychopathology. To unravel the structure of the PANSS items, a considerable number of factor analyses have been performed and most published studies favour a five-factor solution: negative, positive, disorganised (or cognitive), excited and depression/anxiety factors [4,5].

From the clinician's perspective, the PANSS Excited Component (PANSS-EC) is one of the simplest and most intuitive scales used to assess agitated patients [6]. The PANSS-EC consists of 5 items: excitement, tension, hostility, uncooperativeness, and poor impulse control. The 5 items from the PANSS-EC are rated from 1 (not present) to 7 (extremely severe); scores range from 5 to 35; mean scores ≥ 20 clinically correspond to severe agitation [7]. This set of items detects differences between drug and placebo when evaluating acute agitation and aggression in psychiatric patients [5,7-10] with different psychiatric pathologies [7,8,11-18].

Despite its widespread use in research and clinical practice, the PANSS-EC subscale has not been validated against other established rating scales [19], nor for its use in routine practice. Most information about its psychometric properties comes from the global analysis of the PANSS scale. Consequently it is important to know the clinical meaning of its scores in daily clinical practice, outside the restrictions imposed by experimental designs.

This study was designed to validate the PANSS-EC in patients with acute psychosis and agitation through the comparison of PANSS-EC ratings with ratings of the Clinical Global Impression of Severity (CGI-S), the Clinical Global Impression of Improvement (CGI-I) and the Agitation and Calmness Evaluation Scale (ACES), in an unselected sample of 278 patients who received oral psychopharmacological treatment according to standard clinical practice at emergency rooms in Spain.

## Methods

### Subjects and procedures

The study was conducted using data from NATURA, an observational, naturalistic, multicentre, prospective study designed to describe trends in the use of oral antipsychotics and complementary treatments in patients with acute psychotic episodes and agitation seen in emergency departments [20,21]. Study participants were outpatients aged 18 or older with acute psychosis and agitation that according to investigators, required oral psychopharmacological treatment at emergency room units. Treatment was prescribed according to standard clinical practice. Patients who had received treatment with antipsychotics or benzodiazepines within 4 hours prior to initial treatment, required intravenous drugs, had a diagnosis of delirium or dementia, or were participating in any clinical trial, were excluded. Patients admitted to a psychiatric emergency room during duty service of investigators were consecutively enrolled. Patients were observed from the time of admission to the emergency room through discharge or transfer from the psychiatric emergency service. Lack of improvement made reintervention possible. Due to the observational nature of the design all medical interventions performed to control symptoms and agitation followed usual clinical practice. The study was conducted according to the Declaration of Helsinki guidelines and approved by the regulatory authorities of Spain and by each centre's ethics committees.

### Assessments

Demographic and admission data included age, sex, average time from diagnosis to admission, diagnosis at emergency room admission, and initial treatment. At admission into the emergency room, agitated patients were clinically assessed and received usual medical care. If symptoms worsened or remained uncontrolled, an additional pharmacological intervention ("reintervention") was prescribed according to the usual medical practice. Patients could either be discharged home or admitted into hospital. Severity of agitation was assessed according to the PANSS-EC, ACES and CGI-S at admission, before the first reintervention (if any) and at discharge from the emergency room. All three scales were administered at the same three described time points. The improvement of agitation was also assessed by CGI-I before the first reintervention (if any) and at discharge to document the clinical changes that occurred as a result of the pharmacological intervention.

CGI-S and CGI-I scales are well-recognized and established psychometric instruments [22], suitable to measure the severity of agitation and its improvement or worsening compared with the patient's condition at admission. The CGI-S assesses the clinician's impression of the current severity of agitation using scores from 1 (normal, not at all agitated) to 7 (among the most extremely agitated patients). The CGI-I assesses the patient's improvement since the beginning of the study on a 7-point scale ranging from 1 (very much improved) to 7 (very much worse). The CGI has been validated in psychotic, mood and anxiety disorders. It has been confirmed as valid, reliable and sensitive to changes, and presents the required profile for use as a clinical outcome measure suitable for routine use [22,23].

The ACES consists of a single item that rates overall agitation and sedation at the time of evaluation, where 1 indicates marked agitation; 2, moderate agitation; 3, mild agitation; 4, normal behaviour; 5, mild calmness; 6, moderate calmness; 7, marked calmness; 8, deep sleep; and 9, unarousable. This scale has a high convergent validity and high reliability [13,24] and has been used in several clinical trials.

### Statistical methods

#### Validity

According to current trends, measurement or test score validation is an ongoing process wherein one provides evidence to support the appropriateness, meaningfulness and usefulness of the specific inferences made from scores about individuals from a given sample in a given context [25]. As Zumbo BD has pointed out, the feature being validated is the inferences one makes from a measure assuming that inferences made from all empirical measures, irrespective of their apparent objectivity, have a need for validation. Therefore, validity depends on the interpretations and uses of the test results and should be focused on establishing the inferential limits of the assessment, test or measure. Validity statements are not dichotomic (valid/invalid), but rather described on a continuum. They depend upon the cumulative information that several studies have shielded on the topic. Validation practice has also evolved from a fragmented approach to a comprehensive, unified approach in which multiple sources of data are used to support an argument. Validity, then is a unified concept, and validation is a scientific activity based on the collection on multiple and diverse types of evidence [26].

From this perspective, and in order to assess the face validity of the tool, a sample of eight psychiatrists with expertise in treating schizophrenic patients with symptoms of agitation was asked to comment on the PANSS-EC subscale. Psychiatrists were requested to evaluate and provide their overall opinion on a series of questions about the readiness, suitability and feasibility of the instrument. To determine the construct validity, they were also asked about their impression of the importance, frequency and clarity of each item on a 1 to 7 point scale. Correlation (Spearman's) and regression analyses (linear mixed models) as well as equipercentile linking of the CGI-S, ACES and the PANSS-EC items were conducted to examine the scale's diagnostic validity.

The equipercentile linking is defined as a statistical process that is used to adjust scores on test forms so that scores on the forms can be interchangeable [27]. It should be considered when alternate forms of tests exist, scores on the alternate forms are to be compared, and the alternate forms are built to the same detailed specifications so that they are similar to one another in content and statistical characteristics. In the psychometric literature the term "linking" is referred to the search of corresponding points on different, but correlated, measurement devices. Different linking procedures can be found in the literature [28,29], being the equipercentile procedure, the most accurate one. The algorithm of this method is as follows: in the first step, percentile rank functions are calculated for both variables. Using the percentile rank function of one variable and the inverse percentile rank function of the other, we find for every score of one variable a score on the other variable that has the same percentile rank. All these pairs of scores are usually plotted in a graph, and connected by a smooth curve that shows the equipercentile relationship between the two forms. So each point in the graph represents equivalent scores in both tests in the sense that both scores share the same percentile rank in their corresponding distributions.

In the current study we linked the PANSS-EC total score and the CGI-S score as well the PANSS-EC total score and the ACES score at admission to and at discharge from the emergency service. The LEGS statistical programme (version 2.0) provided by The Center for Advanced Studies in Measurement and Assessment of the University of Iowa, College of Education http://www.education.uiowa.edu/casma/index.html and based on the Kolen & Brennan's analysis (2004), has been used. The relation between the CGI-I scale and the percentage PANSS-EC change from admission was also assessed. A principal components factor analysis using equamax rotation was performed to work out the structure of the PANSS-EC items in all patients of the sample and to explore the unidimensionality of the PANSS-EC. The equamax rotation was chosen to be consistent with many previous studies of the PANSS. The factor's extraction was consistent with the eigenvalue ≥ 1 rule.

#### Reliability

Cronbach's alpha determination for measuring the internal consistency of the PANSS-EC and test-retest for analysing its temporal consistency was carried out in all patients. Chronbach's alpha was determined at admission while test-retest was established at admission, before pharmacological reintervention (if any) and at discharge. Two groups of patients were defined according to their clinical state during follow up in the emergency room: 1) those patients who did not show any changes in their overall state of agitation (CGI-I = 4) before the pharmacological reintervention, and 2) those patients who did show changes in their overall state of agitation (CGI-I≠4) before the pharmacological reintervention. Each time the patient was seen after medication had been initiated at admission the clinician compared the patient's overall clinical condition to the one just prior to the initiation of the pharmacological reintervention. The patient's clinical condition was rated on a seven-point scale as follows: "Compared to the patient's condition prior to medication initiation at admission, this patient's condition is: 1 = very much improved since the initiation of treatment; 2 = much improved; 3 = minimally improved; 4 = no change from the initiation of treatment; 5 = minimally worse; 6 = much worse; 7 = very much worse since the initiation of treatment". CGI = 4 was chosen as the cut point measure because it allows for differentiating those patients with clinical changes from those who remained in the same clinical state. It was expected that the CGI-I and the PANSS-EC scores would highly correlate in patients who remained in a similar clinical condition (CGI-I = 4). In contrast, patients whose state of agitation changed significantly following medications given at admission would show lower correlation values with both scales. The intraclass correlation coefficient (ICC) was determined for all cases distinguishing between the two groups of patients: those who required pharmacological reintervention and those who did not. The ICC was calculated for each group. Aditionally, Wilcoxon's signed rank test was applied to compare admission and retest medians. In most studies, to evaluate the reliability and stability of any test, a test-retest comparison procedure is performed. This test-retest comparison can be done by using a paired t-test to compare the mean response in both moments, or by using a Wilcoxon test to compare the medians. Due to the characteristics of the scale used, we have preferred to perform a test-retest analysis by comparing the medians, instead of comparing the means.

#### Responsiveness

For its use in clinical trials, the PANSS-EC should be capable of detecting changes in the clinical condition of the patients that may occur over time, preferably at more than one time-point in order to understand the onset and durability of the effect [30]. In this sense, responsiveness provides additional evidence of the validity of an instrument, and it was measured using the effect size (ES) which gives a continuous parametric measure of the change between admission and follow-up and can be easily interpreted [31-34].

## Results

A total of 278 patients were enrolled in the study (309 screened). The average length of stay at the emergency service before pharmacological reintervention was 2 hours 50 minutes (standard deviation (SD) 4 hours 7 minutes), and a median length of 1 hour 28 minutes. The total average length of stay at the emergency service was 4 hours 23 minutes (SD 6 hours 42 minutes) and a median of 1 hour 53 minutes. A detailed description of sample demographic and clinical characteristics has been published elsewhere [20,21].

### PANSS-EC scores

For all patients (n = 278), the mean PANSS-EC total scores (SD) decreased progressively from 20.38 points (SD 5.07) at entry to 13.07 points (SD 5.45) at discharge. For each item, except for hostility and lack of cooperation, the most frequently reported categories were moderate and fairly severe at admission, and minimum and mild at discharge (Table 1).

**Table 1:** Percentage of patients in each category of the PANSS-EC scale at admission (n = 278), in case of reintervention (n = 106) and at discharge (n = 278)

	Poor impulse control			Tension			Hostility			Lack of cooperation			Excitement		
	**A**	**R**	**D**	**A**	**R**	**D**	**A**	**R**	**D**	**A**	**R**	**D**	**A**	**R**	**D**

**Absent**	0.4	3.7	18.6	0.4	---	13.9	7.2	7.5	33.9	7.2	6.5	31.8	0.4	1.9	19.3

**Minimal**	6.1	7.5	25	1.8	7.5	29.3	14.4	18.7	18.2	9.7	14	19.3	1.8	4.7	26.8

**Mild**	17.6	22.4	28.6	14.7	15	26.8	22.7	17.8	28.9	26.3	16.8	27.1	16.5	19.6	32.1

**Moderate**	40.6	26.2	22.1	36.7	32.7	21.4	28.4	26.2	14.3	25.9	29	12.9	40.3	35.5	17.1

**Moderate-severe**	20.1	30.8	3.9	26.6	31.8	7.5	14.7	16.8	3.6	18.7	16.8	6.8	25.9	23.4	3.2

**Severe**	9.7	6.5	1.8	18	10.3	0.7	9.7	8.4	1.1	8.6	11.2	1.8	12.6	11.2	1.4

**Extremely severe**	1.8	1.9	---	1.8	1.9	0.4	2.9	3.7	---	3.6	4.7	0.4	2.5	2.8	---

PANSS-EC: Excited Component of the Positive and Negative Syndrome Scale; A: admission; R: reintervention; D: discharge

### CGI-S scores

At admission, 62.6% of patients displayed mildly or moderately agitated behaviour. The highest proportion (83.1%) of patients was found to have a CGI-S score in the range of 3 ("mildly agitated") to 5 ("markedly agitated") points. At discharge, 33.2% of patients showed mildly or moderately agitated behaviour while the vast majority (85.7%) of patients had a 1 ("normal, not at all agitated") to 3 ("mildly agitated") points CGI-S score (Table 2).

**Table 2:** Percentage of patients in each category of the CGI-S and ACES scales at admission, in case of reintervention and at discharge

	Admission	Reintervention	Discharge
**CGI-S**			

Normal	0	0.9	39.3

Borderline agitated	10.1	2.8	23.2

Mildly agitated	29.1	12.1	23.2

Moderately agitated	33.5	19.6	10

Markedly agitated	20.5	30.8	3.2

Severely agitated	6.1	22.4	0.7

The most extremely agitated	0.7	11.2	0.4

*ACES*			

Marked agitation	8.3	12.1	1.4

Moderate agitation	49.6	49.5	11.4

Mild agitation	41.0	30.8	35.7

Normal behaviour	0.7	5.6	38.6

Mild calmness	0.4	0.9	8.2

Moderate calmness	---	---	1.4

Marked calmness	---	---	2.1

Deep sleep	---	---	0.7

Not valuable	---	---	0.4

CGI-S: Clinical Global Impression of Severity; ACES: Agitation and Calmness Evaluation Scale

### ACES scores

At admission, 90.6% of patients displayed mild or moderate agitation and at discharge, 47.1% of patients showed mild or moderate agitation (Table 2). Normal behaviour changed from 0.7% at admission to 38.6% of patients at discharge.

A significant number of patients (n = 106, 38.1%) required a pharmacological reintervention at the emergency department. For this subset of patients, at the time of the pharmacological reintervention, the PANSS-EC average score was 20.04 (SD 5.76). The CGI-S scores, on the other hand, showed that 30.8% of the patients were markedly agitated and 22.4% were severely agitated. The CGI-I scores showed that 45.8% of the patients requiring pharmacological reintervention were minimally improved (CGI-I = 3) while 26.2% remained unchanged (CGI-I = 4) at the time of the reintervention (compared to scores at admission). The ACES score showed moderate agitation in 49.5% of the patients and mild agitation in 30.8%.

The Wilcoxon's test showed that the medians change in the agitation score between admission and discharge was statistically significant (p < 0.0001) for all scales: PANSS-EC (-14.54), CGI-S (-13.3) and ACES (-13.02). Changes were also statistically significant in those patients requiring a pharmacological reintervention: PANSS-EC (-5.97), CGI-S (-4.36) and ACES (-4.21). These results showed that the scales detected differences in the state of agitation in most patients between admission and discharge.

#### Validity

Experts found that the scale eased their assessment of the intensity of agitation in patients with acute psychotic episodes, and their follow up. They considered the PANSS-EC useful. The analysis of the importance, frequency and clarity of each individual item on a 5 point scale showed a mean value between 4 and 5 for most items except for clarity in the tension, lack of cooperation and excitement items which showed a 3.33 mean value (SD 0.57).

Spearman's correlation coefficients between the PANSS-EC and the CGI-S scales were r = 0.73 (p < 0.001) at admission and r = 0.8 (p < 0.001) at discharge (n = 278), and r = 0.76 (p < 0.001) amongst those patients requiring a pharmacological reintervention (n = 106). Correlations between PANSS-EC and ACES were r = -0.73 (p < 0.001) at admission, r = -0.71 (p < 0.001) at discharge (n = 278), and r = -0.79 (p < 0.001) amongst those patients requiring a pharmacological reintervention (n = 106). Correlations for the PANSS-EC items varied between 0.64 for lack of cooperation and 0.26 for excitement (p < 0.01) between admission and discharge.

**Figure 1:**
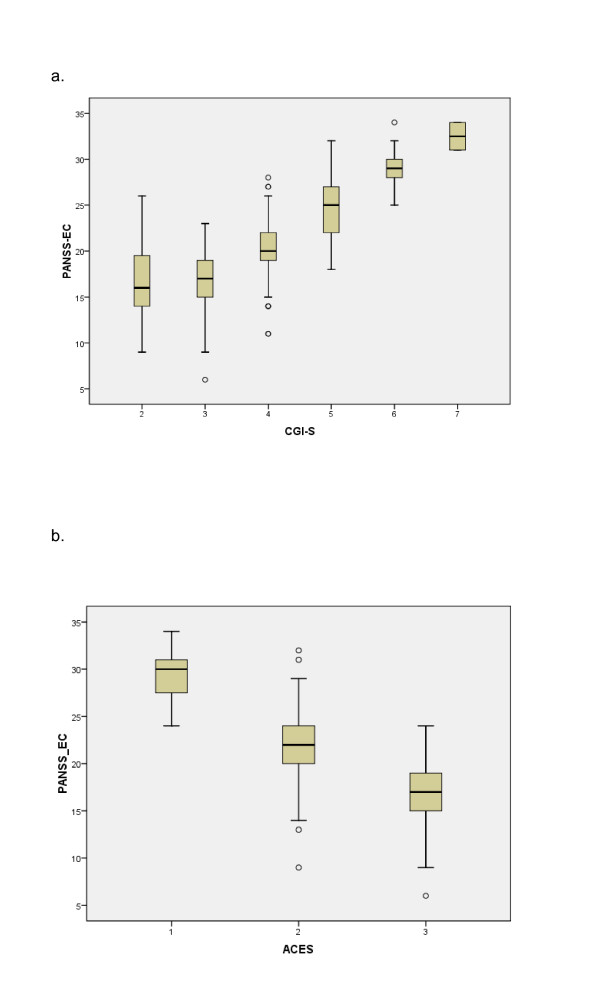
**a. Distribution of the PANSS-EC total scores at patient's admission corresponding to CGI-S values for all patients (unadjusted data)**. Box = 25% and 75% quartiles, line = median, whiskers = minimum and maximum values, circles = outliers. *Note: no participants gave a score of 1 in the CGI-S at admission*. PANSS-EC: Excited Component of the Positive and Negative Syndrome Scale; CGI-S: Clinical Global Impression of Severity. **b**. Distribution of the PANSS-EC total scores at patient's admission corresponding to ACES values for all patients (unadjusted data). Box = 25% and 75% quartiles, line = median, whiskers = minimum and maximum values, circles = outliers. PANSS-EC: Excited Component of the Positive and Negative Syndrome Scale; ACES: Agitation and Calmness Evaluation Scale.

**Figure 2:**
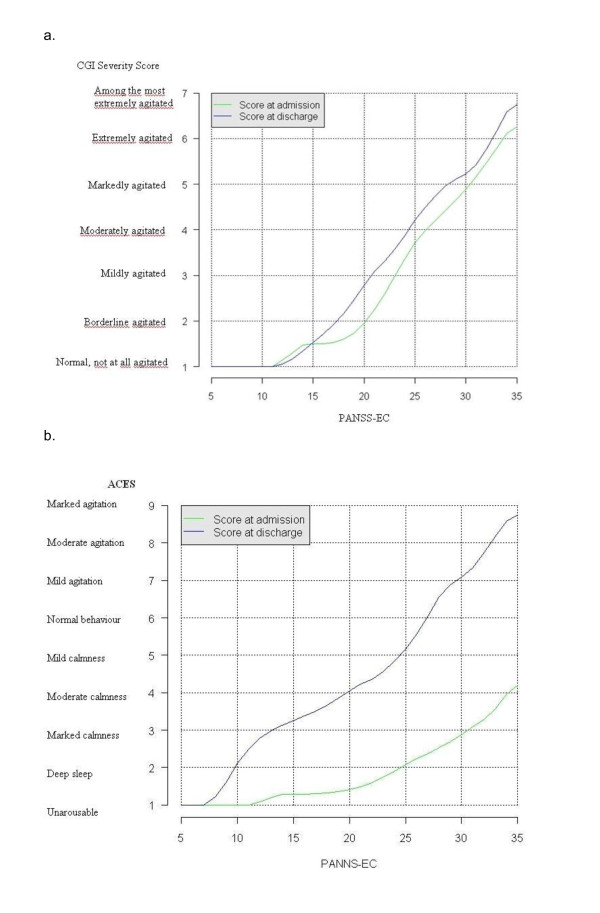
**a. Linking of CGI-S with the PANSS-EC score at admission (green line) and at discharge (blue line)**. The graph plots the corresponding (real) CGI score for every (integer) PANSS-EC score. For the reverse direction, the intersection of the lines indicates an integer CGI value with the graph providing the corresponding PANSS-EC score. PANSS-EC: Excited Component of the Positive and Negative Syndrome Scale; CGI-S: Clinical Global Impression of Severity. **b**. Linking of ACES with the PANSS-EC score at admission (blue line) and at discharge (green line). The graph plots the corresponding (real) ACES score for every (integer) PANSS-EC score. For the reverse direction, the intersection of the lines indicates an integer ACES value with the graph providing the corresponding PANSS-EC score. PANSS-EC: Excited Component of the Positive and Negative Syndrome Scale; CGI-S: Clinical Global Impression of Severity.

At admission, the PANSS-EC and CGI-S were found to be linearly related, with an average increase of 3.4 points (p < 0.0001) on the PANSS-EC for each additional CGI-S point (Figure 1a). At discharge, the relationship between the PANSS-EC and CGI-S was also found to be linear with an average increase of 3.7 points (p < 0.001) on the PANSS-EC for each additional CGI-S point. In a linear model, the CGI-S score explained 66.7% of the variance of the PANSS-EC total score for all patients. Both questionnaires were measured with random error and results were presented in a categorical scale. Considering that a regression analysis usually requires a normal distribution of the data and assumes linearity, in this study, the equipercentile linking was also represented to find out concordance as well as prediction amongst data, and to achieve more comparable scores [35]. The PANSS-EC and CGI-S score at admission and at discharge were linked and presented (Figure 2a). CGI scores were linked to PANSS scores at admission: 1 = 5-11, 2 = 12-14, 3 = 15-19, 4 = 20-23, 5 = 24-27, 6 = 28-32. The PANSS-EC and ACES were found to be linearly and inversely related, with an average decrease of 5.5 points (p < 0.0001) on the PANSS-EC for each additional ACES point (Figure 1b). Using the equipercentile linking method, the poor sensitivity of the ACES scale and its poor capacity for discriminating values that imply sedation (ACES = 5 to 9) seems evident as well as its tendency to a ceiling effect for agitation scores in patients admitted to emergency rooms (Figure 2b). However, the small percentage of markedly sedated patients (ACES ≥ 7) at discharge makes it difficult to guarantee the sensibility of the ACES in this sample.

**Figure 3:**
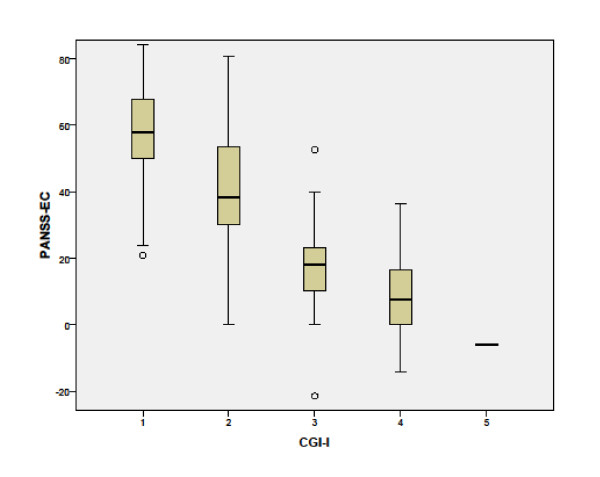
**Distribution of the percentage of reduction in the PANSS-EC score corresponding to CGI-I values from baseline to discharge for all patients (unadjusted data)**. Box = 25% and 75% quartiles, line = median, whiskers = minimum and maximum values, circles = outliers. PANSS-EC: Excited Component of the Positive and Negative Syndrome Scale; CGI-S: Clinical Global Impression of Severity.

The relationship between the PANSS-EC percentage change from admission and CGI-I score at discharge was inverse and linear, with a decrease of 17.98 points (p < 0.001) on the PANSS-EC for each additional CGI-I point (Figure 3). To estimate these ratios the minimal value of 5 was subtracted. The CGI-I score explained 4.6% of the variance (CGI-I ratings of 6 and 7 were not included because of under-representation). Ratings of very much improved corresponded to median reduction of 58% on PANSS-EC; ratings of much improved corresponded to median reduction of 38% on PANSS-EC; and ratings of minimally improved corresponded to median reduction of 18% on PANSS-EC.

The factor analysis resulted in one factor being retained according to eigenvalue ≥ 1 criteria. The variance explained by the factor was 64.43% and the five items exceeded the loading 0, 74. The correlation matrix is represented in Table 3. These findings confirmed the unidimensinality of the PANSS-EC.

**Table 3:** Correlation matrix of the PANSS-EC scale

	Poor impulse control	Tension	Hostility	Uncooperativeness	Excitement
Poor impulse control	1.000				

Tension	0.517	1.000			

Hostility	0.603	0.602	1.000		

Uncooperativeness	0.647	0.504	0.649	1.000	

Excitement	0.546	0.576	0.448	0.451	1.000

PANSS-EC: Excited Component of the Positive and Negative Syndrome Scale

#### Reliability

Cronbach's alpha coefficient was 0.86. Before pharmacological reintervention, when psychiatrists reported no changes on patient's agitation state, the Intraclass Correlation Coefficient (ICC) was 0.9 (PANSS-EC total score), and before discharge from the emergency room, when psychiatrists reported no changes on patient's agitation state (ICG-I = 4, n = 17), ICC was 0.8 Due to the limitations of this measurement, we can only estimate the reliability through the ICC on those patients whose true score does not change over the time period analyzed, i.e. in the group of patients where CGI = 4. In a recent papers, Laenen A and Alonso A [36,37] proposed a new measurement for reliability of a rating scale, based on the classical definition of reliability, as the ratio of the true score variance and the total variance, which is estimated from the covariance parameters obtained from a linear mixed model. As we have just fitted a classical linear regression model, we will take into account this measurement in future works.

#### Responsiveness

The magnitude of the change in PANSS-EC scores between patients' admission and discharge from the emergency service was large (ES = 1.44); it was smaller between patients' admission and reintervention (ES = 0.46). The PANSS-EC was capable of detecting changes of different magnitude at different time-points. As expected, the magnitude of the change in the agitation state of patients was larger from admission to discharge than from admission to follow up in the emergency room when a pharmacological intervention was needed.

## Discussion

The PANSS-EC is a commonly used instrument, to assess severely aggressive and agitated patients; however, it has not yet been validated against other recognized scales. According to the authors' best knowledge, this is the first article reporting a specific validation of the PANSS-EC as an instrument independent from the PANSS scale and against established rating scales such as the CGI-S or the ACES [13].

Several studies have assumed PANSS-EC validity based on data from the original PANSS study conducted by Kay et al. (1987) and used in multiple trials [10,12,14,38]. Huber et al. (2008) [39], for instance, carried out a validation study of the Clinical Global Impression Scale for Aggression (CGI-A) in psychiatric patients seen in the emergency room using the PANSS-EC subscale as the comparative instrument. The CGI-A has been derived from the CGI-S scale which was designed as an overall measure of illness severity in psychiatric disorders. The CGI-A specifically measures aggression rather than allowing for a global assessment of the psychiatric state of patients.

Most of the studies that have explored the factorial of the PANSS are based on data coming from clinical trials. In the present study, we used data from an observational study in patients with acute psychotic episodes and agitation who entered the emergency service, a sample of patients treated in routine clinical practice settings.

The factorial analysis confirms the unifactorial structure of the PANSS-EC subscale with the five suggested items. The variance, explained as the matrix of components, confirms the robustness of the separated use of the excitement component of the PANSS. The Cronbach's alpha coefficient was higher than the established standards and superior to other coefficients reported in recent studies analysing factorial structure of the whole PANSS [5]. Being a unidimensional and consistent tool with highly correlated scores, the PANSS-EC allow for acceptably assessing agitated patients. Another report [6] identifies a cluster of mania-like symptoms through the use of PANSS-based factor analysis of data pooled from three patient samples. This factor shows good internal reliability. That report, however, only considers four items and leaves out the tension item that has a higher weight in the depression subscale.

The ICC informs about the desirable behaviour of the scale considering that the internal consistency is higher when the state of agitation of patients does not change in an opposed way. The sensitivity of the scale assessed through the floor and ceiling effect is adequate. Less than 7.2% of the patients reported the minimum score and 3.5% the maximum score. The correlation between PANSS-EC and CGI-S total scores was high (r = 0.73-0.83). Correlations between the PANSS-EC and the ACES scales were equally high (r = -0.73, -0.71). These results are similar to those reported by other authors. For instance, Huber et al. (2008) found correlations between the CGI-S and the PANSS-EC scales of 0.83; Meehan et al. (2002) reported an r = -0.71 between the PANSS-EC and the ACES scales; Leucht et al. (2005) [40] reported coefficients of 0.56 and 0.73 between the PANSS-EC and CGI-S scales. Using the entire PANSS, Levine et al. (2008) found correlations of r = 0.61 to r = 0.73 between the same scales. The ACES specificity for measuring agitation in psychiatric patients explains the ceiling effect found in this study of agitated patients.

Parallelism between the study by Huber et al. (2008) and ours is worth noting. In both studies there is a linear relation between the two instruments as well as an increase in the scoring of the PANSS-EC for each point considered of the CGI-S scale. While our results show that scores increase 3.4 points, Huber's study reports 4.6. However the increase estimates are not directly comparable between studies, because they used a CGI-S version with five levels of responses while we used the original version of seven options.

The responsiveness result that we have obtained is excellent and provides additional evidence of the validity of PANSS-EC. One of the most interesting findings of the validation process of the PANSS-EC subscale has been the quantification of the reductions on the scoring system of the scale, which correlates well with states of agitation, such as minimally improved (18%), much improved (38%) and very much improved (58%). These similarities with the CGI-I scale suggest an improvement in patients' agitated state and they could be taken as the minimum clinically significant differences.

### Strengths and limitations

The large sample study of psychotic patients with an episode of agitation contributes to the external validity of these results. Analysis shows that this is an adequate and useful instrument for assessment of agitated and aggressive patients. Limited ceiling effects are unlikely to limit the generalizability of results, since PANSS-EC showed a strong linear correlation with well-known rating scales such as CGI-S and ACES (particularly with the ACES). PANSS-EC has also shown an excellent capacity to detect real changes in agitated patients. Changes in percentages represent improvements in health status that can be detected, measured and confirmed. In order to overcome methodological concerns against linear regression analysis and equipercentile linking, we use both to assess the relation amongst the PANSS-EC, the CGI-S and the ACES scales.

The short follow-up period is amongst the main study limitations. Given the naturalistic character of the study, we have focused on the time patients stay in the emergency service, which is usually very short. This brief follow-up period may have possibly influenced the test-retest reliability. Nevertheless, the ES test offers a very good result, showing that the instrument holds a great sensitivity to changes. Intermediate assessments of those patients requiring pharmacological reintervention have been conducted very shortly after admission, and changes in the state of patients' agitation may not be significant enough as to find differences. Another possible study limitation is a treatment bias. We excluded patients on intravenous medications because many of them frequently perceive the intravenous route to be compulsory. These perceptions may negatively affect the patient-doctor relationship and may have some bearing on treatment adherence and follow-up by restraining patients' contribution to the therapeutic plan [21].

It is important to mention the conceptual barriers when referring to agitation and aggression. Agitation is still a poorly understood phenomenon. The absence of a clear definition of the syndrome is associated with problems to measure it. Agitation may appear in the context of almost any severe psychiatric disorder, and its features may vary greatly according to the underlying condition. Moreover, cultural differences have also been suspected of producing significant differences in the display of agitation. These features, which are inherent to the disease being explored, together with the design of the study (observational) and the type of patients (agitated) being assessed, make it highly improbable to avoid all possible bias. Furthermore, in our study, the same clinician assessed each patient's agitation using different scales. This may have led to overestimate the statistical correlations.

## Conclusions

Despite the wide use of the PANSS-EC scale, a validation study to inform on its psychometric properties was missing. The goal of this study has mainly focused on filling in this gap. The present results show PANSS-EC has a good sensitivity; without either ceiling or floor effect; with an acceptable Cronbach's alpha and an optimal temporal stability. The factorial analysis has revealed a unifactorial structure and the responsiveness has shown excellent results. These results are even more significant if the short period of time that patients stayed in emergency room is taken into account.

## Competing interests

The study was sponsored by Lilly.

Alonso Montoya and Amparo Valladares work at Lilly. Luis San and Rodrigo Escobar work at different psychiatric services in Spain. Luis Lizán and Silvia Paz work at Outcomes'10, an independent research group.

## Authors' contributions

All authors contributed to the development of the protocol and to the collection and/or analysis of data for this study. All authors drafted and/or critically read and revised the manuscript for important intellectual content and have approved the final manuscript for publication.
